# Role of miR-455-3p in the alleviation of LPS-induced acute lung injury by allicin

**DOI:** 10.1016/j.heliyon.2024.e39338

**Published:** 2024-10-12

**Authors:** Yueliang Zheng, Gaoxiang Li, Aili Shi, Junping Guo, Yingge Xu, Wenwei Cai

**Affiliations:** a Emergency and Critical Care Center, Department of Emergency Medicine, Zhejiang Provincial People's Hospital (Affiliated People's Hospital), Hangzhou Medical College, Hangzhou, Zhejiang, China; b Rainbowfish Rehabilitation & Nursing School, Hangzhou Vocational & Technical College, Hangzhou, Zhejiang, China

**Keywords:** Acute lung injury, Allicin, miR-455-3p, Claudin-4

## Abstract

Acute lung injury (ALI) is a type of diffuse lung injury that seriously affects the survival of critically ill patients. MicroRNAs (miRNAs) can serve as promising therapeutic targets or offer insights for the development of potential therapeutic strategies against ALI. In our previous study, we demonstrated the protective effect of allicin in ALI, but the role of miRNAs in the alleviation of ALI by allicin remains unclear. This study aimed to investigate whether miRNAs mediate the effects of allicin on ALI. Cell viability and proliferation were determined using CCK-8 and EdU assays, respectively, while cellular apoptosis was analyzed by flow cytometry. The claudin-4 protein was detected by quantitative real-time reverse-transcription polymerase chain reaction (qRT-PCR) and western blotting. The binding of miR-455 with claudin-4 was determined by bioinformatics analysis and validated by dual luciferase reporter assays. The lung wet/dry ratio of lipopolysaccharide (LPS)-treated rats was determined by hematoxylin and eosin (HE) and TUNEL staining of the pulmonary tissues. The levels of myeloperoxidase (MPO), interleukin (IL)-2, IL-6, and tumor necrosis factor (TNF)-α were determined by enzyme-linked immunosorbent assay (ELISA). We observed that allicin alleviated LPS-induced injury in A549 cells, and claudin-4 knockdown reversed the protective effect of allicin in ALI. Claudin-4 is a direct target of miR-455-3p, and miR-455-3p overexpression partially reversed the protective effect of allicin in LPS-treated A549 cells. Subsequent *in vivo* experiments confirmed that allicin protects against LPS-induced ALI by regulating the miR-455-3p/claudin-4 axis. The study revealed that the protective effect of allicin in ALI is mediated via miR-455-3p, which suppresses the expression of claudin-4.

## 1 Introduction

Acute lung injury (ALI) is a type of diffuse lung injury caused by pneumonia and other factors, and is characterized by pulmonary edema, respiratory distress, and hypoxemia, which later develops into the more severe acute respiratory distress syndrome (ARDS) [[1], [2], [3]]. ALI/ARDS is one of the main causes of morbidity and mortality in critically ill patients; however, there is a scarcity of effective clinical treatments for ALI and ARDS to date [4]. Certain intervention strategies, including lung-protective mechanical ventilation and fluid-restrictive resuscitation strategies, can improve patient outcomes [5]. However, the morbidity and mortality rates of ALI remain high even after the application of these intervention strategies [6]. It is therefore necessary to explore and establish novel approaches for the treatment of ALI.

MicroRNAs (miRNAs) are approximately 18–23 nucleotides in length and are involved in the post-transcriptional regulation of gene expression [7]. Previous studies have demonstrated that miRNAs play crucial roles in ALI [8]. For instance, miR-27a is downregulated in the lung tissues of lipopolysaccharide (LPS)-challenged mice, and the overexpression of miR-27a alleviates LPS-induced ALI in mice [9]. In contrast, Zhang et al. reported that the inhibition of miR-762 significantly attenuates the LPS-mediated impairment of A549 lung cells [10]. The study also demonstrated that Shikonin improves sepsis-induced lung injury by regulating the miRNA-140-5p/TLR4 axis *in vitro* and *in vivo* [11]. These findings indicate that miRNAs can serve as promising therapeutic targets or offer insights for the development of potential therapeutic strategies against ALI.

Allicin is an active ingredient of garlic with numerous biological activities. Previous studies have reported that allicin possesses various biological functions, including anti-microbial, anti-tumor, anti-oxidant, and anti-inflammatory properties [[12], [13], [14], [15]]. Allicin also protects against ischemia/reperfusion (I/R) injury and prevents cardiovascular diseases [[16], [17], [18]]. Recent studies have demonstrated the protective effect of allicin in lung injury [19,20]. In our previous study, we demonstrated that allicin protects against LPS-induced ALI by upregulating the expression of claudin-4 [21]. However, few studies have explored the relationship between miRNAs and the function of allicin in alleviating ALI. Liu et al. demonstrated that allicin protects against myocardial I/R injury by accelerating angiogenesis via the miR-19a-3p/PI3K/AKT axis [16]. Xie et al. reported that allicin inhibits the growth of osteosarcomas by promoting oxidative stress and autophagy by inactivating the MALAT1 lncRNA-mir-376a-wnt/β-catenin signaling pathway [22].

Claudin-4 is a tight junction protein that plays an important role in ALI [23]. Using animal experiments, Dai et al. observed that ventilation-induced lung injury and pulmonary edema are associated with the degradation of claudin-4 and occludin [24]. Jacob et al. observed that a reduction in the expression of claudin-4 disrupts lung epithelial barrier function, which leads to the onset of pulmonary edema [25]. By analyzing sections of lung tissues from rats, a previous study demonstrated that ischemia/reperfusion (I/R)-induced lung injury reduces the expression of claudin-4, occludin, and ZO-1; however, treatment with drugs that upregulate the expression of claudin-4 reverses the effects of I/R injury [26]. Our previous studies have also confirmed that the levels of claudin-4 decrease after lung injury [27], and that the upregulation of claudin-4 can alleviate lung injury [28]. Using the TargetScan database, we previously identified that miR-455-3p can bind to claudin-4. We therefore speculated that miR-455-3p might mediate the protective effect of allicin against lung injury. The present study therefore aimed to investigate whether the protective effect of allicin against ALI is mediated via miR-455-3p.

## 2 Materials and methods

### 2.1 Cell culture and treatment

The human lung adenocarcinoma A549 epithelial cell line was purchased from American Type Culture Collection (ATCC; Manassas, VA, USA) and cultured in Dulbecco's modified Eagle medium (DMEM, Thermo Fisher Scientific, Waltham, MA, USA) supplemented with 10 % fetal bovine serum (FBS, Thermo Fisher Scientific) at 37 °C in a humidified atmosphere of 5 % CO_2_ and 95 % air. The optimal concentration of LPS was determined by treating the cells with various concentrations of LPS (0, 2.5, 5, 10, 20, and 40 μg/mL; Sigma-Aldrich, St. Louis, MO, USA). The cells were subsequently treated with 10 μg/mL LPS by incubating for 6 h, following which allicin (Selleck Chemicals, Houston, TX, USA) was added to the culture medium. The optimal concentration of allicin was similarly determined by treating the LPS treated-A549 cells with different concentrations of allicin (0, 5, 10, 20, or 40 μl/mL) for 48 h. The A549 cells in the control group were treated with PBS.

### 2.2 CCK-8 assay

The LPS-induced cells were plated in a 96-well plate at the density of 5 × 10^3^ cells/well, and subsequently exposed to allicin at indicated concentrations. Cell viability was measured using a CCK-8 kit (Dojindo Laboratories, Japan). Then, 10 μl of CCK-8 was added to the well at the indicated times, and the cells were incubated for 2 h at 37 °C. The optical density (OD) was subsequently measured at 450 nm using a Microplate Reader.

### 2.3 Cell transfection

MiR-455-3p mimics and miRNA-negative control (miR-NC) were purchased from RiboBio Co., Ltd. (Guangzhou, China). SiRNAs targeting claudin-4 and the NC-siRNA were designed and synthesized by GenePharma Co. Ltd. (Shanghai, China). The A549 cells were transfected with Lipofectamine 2000 (Invitrogen, Carlsbad, CA, USA) at a confluence of approximately 80 %, according to the manufacturer's instructions.

### 2.4 Edu assay

The experimental protocols of the Edu assays for measuring cell proliferation have been reported previously [28].

### 2.5 Measurement of apoptosis

Apoptosis was measured using a FITC Annexin V apoptosis detection kit I (BD Biosciences), according to the manufacturer's instructions. The cells harvested in 500 μl binding buffer were stained with 5 μl Annexin V-APC and 5 μl propidium iodide (PI) by incubating for 15 min in the dark at room temperature. The cells were then analyzed by flow cytometry (Becton & Dickinson Company, Franklin Lakes, NJ, USA) and the data were evaluated using the FlowJo software.

### 2.6 Western blotting

The experimental protocols for western blotting have been reported previously [28]. The proteins were separated by sodium dodecyl sulfate polyacrylamide gel electrophoresis (SDS-PAGE) using 10 % gels, electro-transferred to a polyvinylidene fluoride (PVDF) membrane (Millipore), probed with the primary antibody against claudin-4 (16195-1-AP, Proteintech Group Inc., CA, USA), and detected by chemoluminescence (GE Healthcare Life Sciences, Chalfont, UK). The images were subsequently captured using the ImageLab software (Bio-Rad, CA, USA). The protein expression levels were normalized to the levels of β-actin protein.

### 2.7 Quantitative real-time reverse-transcription polymerase chain reaction (qRT-PCR)

The total RNA was extracted by lysing cells with Trizol reagent (Takara, Dalian, China). For miRNA quantification, complementary DNA (cDNA) was synthesized using a Mir-X miRNA First-Strand Synthesis Kit (Takara). For mRNA quantification, cDNA was synthesized using a PrimeScript ^RT^ RT reagent Kit with gDNA Eraser (Perfect Real Time; Takara). qRT-PCR was performed with SYBR Green Mix (Takara) using an ABI 7500 system (Applied Biosystems; Thermo Fisher Scientific, Inc.). U6 or GAPDH was used as the loading control. The primers for qRT-PCR were synthesized by Shanghai Sangon (Shanghai, China), and the sequences of the forward (F) and reverse (R) primers are provided hereafter:

Claudin-4-F: 5′-TGAAAGCGCCAAGGCCAAGA-3′

Claudin-4-R: 5′-GCAGAGCGGGCAGCAGAATA-3′

### 2.8 Dual-luciferase reporter assays

The interaction between miR-455-3p and the 3′-unranslated region (UTR) of claudin-4 mRNA was predicted using the TargetScan database (http://www.targetscan.org/vert_72/). The 3′-UTR of claudin-4 containing the binding region of miR-455-3p and the mutated region were cloned into a pGL3 luciferase vector obtained from Promega (Madison, WI, U.S.A.) to generate the WT claudin-4 3′-UTR reporter and MUT claudin-4 3′-UTR, respectively. The WT and MUT claudin-4 3′-UTR reporters were separately cotransfected with miR-455-3p mimics or miR-NC using Lipofectamine 2000 (Invitrogen, Carlsbad, CA, USA). After 48 h, the cells were analyzed using a Dual-Luciferase Reporter Assay System (Promega, Madison, WI, USA), according to the manufacturer's instructions, for detecting the relative activity of luciferase.

### 2.9 Animals and ALI model

Sprague-Dawley rats, weighing 200 g and aged 6 weeks, were purchased from Weitonglihua (Beijing, China) and maintained in the animal facility of the First Affiliated Hospital, School of Medicine, Zhejiang University, under pathogen-free conditions. All the procedures were approved by the Ethics Committee of the First Affiliated Hospital of Zhejiang University, in accordance with the Guide for Care and Use of Laboratory Animals of the National Institutes of Health (NIH Publications, No. 8023, revised 1978) and the ARRIVE guidelines [29]. In order to determine the survival rate, the rats were randomly divided into four groups, namely, the Sham group, ALI model group, ALI + allicin group, and ALI + allicin + agomiR-455 group, with each group consisting of a total of 10 animals. The rats in the sham group only received normal saline, while the rats in the ALI model group received 5 mg/kg LPS by airway administration. The rats in the ALI + allicin treatment group received 10 mg/kg allicin via tail vein injections for 7 days after the establishment of the ALI model. Agomir-455-3p was synthesized by RiboBio Co., Ltd., Guangzhou, China. The rats in the ALI + allicin + agomiR-455 group received agomir-455-3p via tail vein injections on every third day until the induction of ALI as previously described. The survival of the rats in each group was monitored on a daily basis. The rats were humanely sacrificed by an overdose of anesthesia after 7 days. The role of mir-455 in the protective effect of allicin against ALI was investigated using 6 animals from each group. The rats were sacrificed after 3 days and the samples were collected for subsequent analyses.

### 2.10 Lung water content and lung W/D weight ratio of LPS-treated rats

The thoracic cavity of the rats was opened after sacrifice and the whole lung was immediately dissected out. The lung was collected, weighed, and dried at 80 °C for 48 h, and subsequently reweighed for calculating the lung water content and lung W/D weight ratio. The lung water content was calculated using the following formula: ((wet weight of lung - dry weight of lung)/(wet weight of lung)) × 100.

### 2.11 Myeloperoxidase (MPO) activity assay

The lung tissues were weighed and homogenized, and the supernatant was used for the MPO activity assay (Nanjing Jiancheng Bioengineering Institute, Nanjing, China) based on the manufacturer's instructions. The absorbance was determined at 460 nm.

### 2.12 Collection of bronchoalveolar fluid (BALF)

The BALF was collected by flushing the lungs thrice with 1 mL cold sterile physiological saline (0.9 % NaCl) via the tracheal cannula. The BALF was centrifuged at 1500 rpm for 10 min at 4 °C, following which the supernatant was collected and stored in a freezer at −80 °C until further use.

### 2.13 Enzyme linked immunosorbent assay (ELISA)

The levels of IL-6, IL-2, and TNF-α in the BALF of the rats were determined using an ELISA kit (Dakewe, Beijing, China), according to the manufacturer's protocol.

### 2.14 Wright's-Giemsa staining

The number of white blood cells was enumerated using a Wright's-Giemsa staining kit (D010-1, Nanjing Jiancheng Bioengineering Institute, Nanjing, China), according to the manufacturer's protocol.

### 2.15 Histological examination

For assessing lung injury, the right middle lobes of the lung tissues were immersed in 4 % paraformaldehyde for 24 h, embedded in paraffin, sliced into 4 μm-thick sections, and stained with HE for 15 min at room temperature. The tissue sections were subsequently observed under a microscope. The severity of ALI was assessed using a semi-quantitative histologic scoring system described in a previous study [30]. In this system, the assigned scores range from 0 (indicating no lesions) to 4 (indicating major and extended lesions), depending on the severity of the lesions. The criteria for each score were clearly defined, including the occurrence of alveolar necrosis, vascular congestion, and the infiltration of neutrophils and macrophages.

### 2.16 TUNEL assay

In order to assess the degree of cellular apoptosis, the right middle lobes of the lung tissues were dewaxed, rehydrated, and digested with proteinase K for 30 min. After washing with PBS, the TUNEL assay was performed according to the protocol described in the TUNEL Apoptosis Detection Kit (Alexa Fluor 488; Roche, Basel, Switzerland).

### 2.17 IHC-P staining

The right middle lobes of the lung tissues were embedded in liquid paraffin and sliced into 4 μm-thick sections. The slices were immersed in xylene for 15 min and rehydrated in water with an ethanol gradient, following which the sections were immersed in citric acid (pH 6.0) for 10 min. After cooling to room temperature, the sections were washed with water and PBS buffer for 15 min. The sections were subsequently incubated with 3 % H_2_O_2_ for 10 min and blocked with 5 % BSA for 30 min. The sections were then incubated overnight with the primary antibody against claudin-4 (16195-1-AP, Proteintech Group Inc., CA, USA) at 4 °C, and subsequently incubated with the corresponding secondary antibodies at 37 °C for half an hour. The sections were finally stained with a DAB plus kit according to the manufacturer's protocol.

### 2.18 Statistical analyses

All the results were analyzed using GraphPad Prism 5, and presented as the mean ± standard deviation (SD), displayed a normal distribution. The data were analyzed by Student's t-test or one-way analysis of variance (ANOVA) and Tukey's test. The Kaplan-Meier method was used to calculate the survival rates, and the log-rank test was performed for comparing the survival distributions. *P* < 0.05 was considered to be statistically significant.

## 3 Results

### 3.1 Allicin alleviates LPS-induced injury in A549 cells

**Fig. 1:**
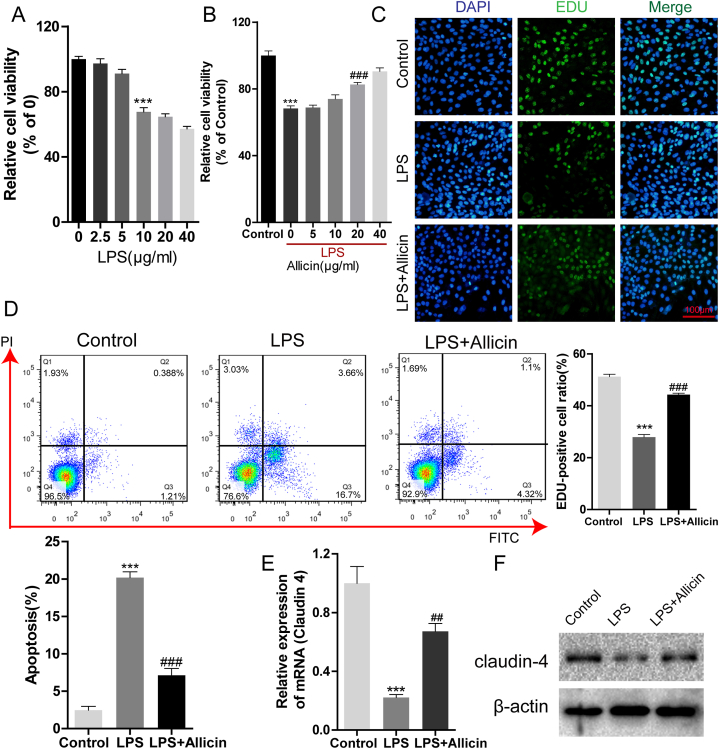
Allicin enhanced the proliferation and inhibited the apoptosis of LPS-treated A549 cells. (A) CCK-8 assays were performed for measuring the cytotoxicity of LPS in A549 cells. (B) The viability of A549 cells treated with different concentrations of allicin (0, 5, 10, 20, and 40 μg/mL) following exposure to LPS was determined by CCK-8 assays. (C) The proliferation of A549 cells treated with 20 μg/mL allicin following exposure to LPS was determined by EdU assays (magnification: 200 × ). (D) The apoptosis of A549 cells treated with 20 μg/mL allicin following exposure to LPS was measured by flow cytometry. The expression of claudin-4 mRNA (E) and protein levels (F) were determined by qRT-PCR and western blotting, respectively. ∗∗∗*P* < 0.001 vs. control; ##*P* < 0.01, ###*P* < 0.001 vs. LPS. The original Western blot bands are shown in Fig. S2 in the supplemental file.

We initially evaluated the effect of allicin on LPS-induced ALI *in vitro*. The cytotoxicity of LPS to A549 cells was assessed using cell counting kit-8 (CCK-8) assays, and the findings revealed that the viability of A549 cells was affected when the dose of LPS was higher than 10 μg/mL (*P* < 0.0001; Fig. 1A). The subsequent experiments were therefore performed with 10 μg/mL LPS. As depicted in Fig. 1B, cell viability decreased significantly following treatment with LPS, while treatment with allicin at concentrations of 20 μg/mL and higher suppressed the LPS-induced reduction in cell viability (*P* < 0.0001; Fig. 1B). The subsequent experiments were therefore performed with 20 μg/mL allicin. The results of EdU assays demonstrated that exposure to LPS distinctly reduced DNA synthesis in A549 cells compared to that of the control group, while treatment with allicin reversed the effect of LPS (*P* = 0.0001; Fig. 1C). Flow cytometric analyses also revealed that allicin prevented the LPS-induced apoptosis of A549 cells (*P* = 0.0004; Fig. 1D). Altogether, these findings indicated that allicin could protect against ALI *in vitro*.

### 3.2 Claudin-4 knockdown reversed the protective effect of allicin

We have previously demonstrated that claudin-4 plays a role in the development of ALI [28,31]. In this study, we determined the association between claudin-4 and allicin *in vitro*. As expected, exposure to LPS decreased the expression of claudin-4, while treatment with allicin increased claudin-4 expression (*P* = 0.0015; Fig. 1E and F). We subsequently investigated the relation between allicin and the expression of claudin-4 by applying a claudin-4 small interfering RNA (siRNA). The results demonstrated that claudin-4 knockdown suppressed the increase in cell viability and proliferation induced by allicin in LPS-treated A549 cells (Figs. S1A and 1B). Moreover, the expression of claudin-4 was elevated by allicin but reversed by the co-application of allicin and si-claudin4 in the presence of LPS (Figs. S1C and 1D). These results indicated that allicin played a protective effect in ALI by increasing the expression of claudin-4.

### 3.3 Claudin-4 is a direct target of miR-455

**Fig. 2:**
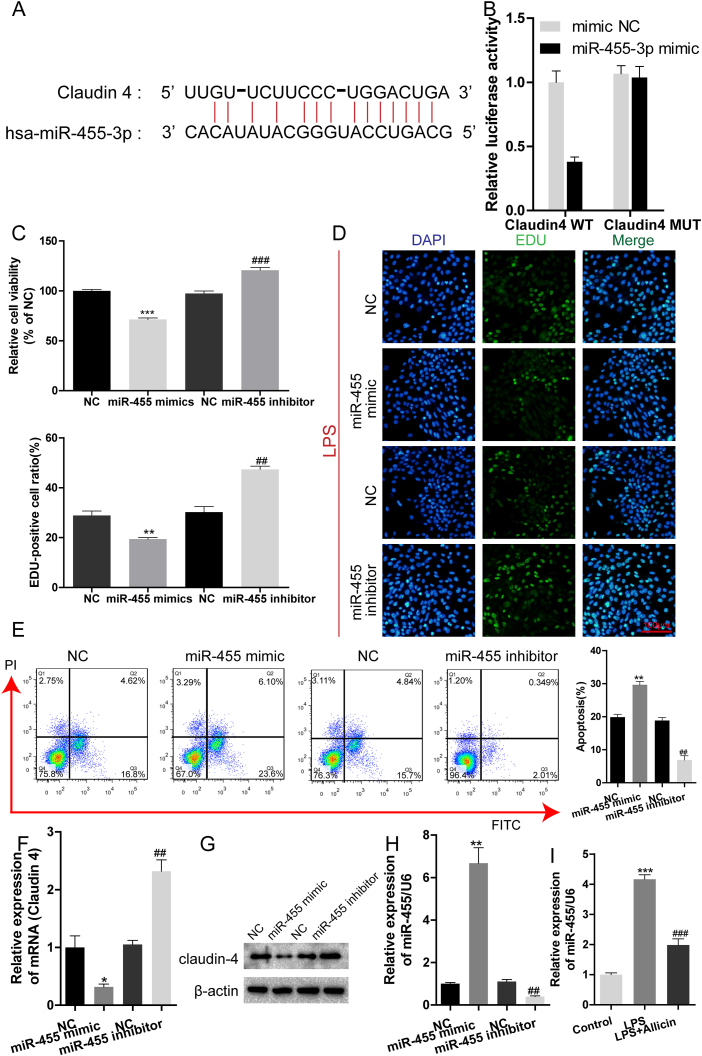
Claudin-4 is a direct target of miR-455. (A) The predicted binding sequence of miR-455 in the 3′-UTR of claudin-4. (B) The activity of luciferase was determined by dual-luciferase reporter assays. (C) The viability of A549 cells following transfection with NC, miR-455 mimics, or miR-455 inhibitors was determined by CCK-8 assays. (D) The proliferation of A549 cells following transfection was determined by EdU assays (magnification: 200 × ). (E) The apoptosis of A549 cells following transfection was determined by flow cytometry. The expression of claudin-4 mRNA (F) and protein levels of claudin-4 (G) following the transfection A549 cells was determined by qRT-PCR and western blotting, respectively. (H) The expression of miR-455 following the transfection of A549 cells was determined by qRT-PCR. (I) Expression of miR-455 in A549 cells treated with allicin following LPS exposure. ∗*P* < 0.05, ∗∗*P* < 0.01, ∗∗∗*P* < 0.001 vs. NC; ##*P* < 0.01, ###*P* < 0.001 vs. NC. The original Western blot bands are shown in Fig. S3 in the supplemental file.

The prediction results from the TargetScan database revealed the presence of a binding site between miR-455-3p and claudin-4 (Fig. 2A). The results of the dual-luciferase reporter assay revealed that the activity of luciferase was lower in A549 cells co-transfected with the miR-455-3p mimic and claudin-4-WT compared to that of A549 cells co-transfected with miR-NC and claudin-4-WT; however, there were no obvious changes in the activity of luciferase between the group treated with mimic NC and claudin-4-MUT and the group treated with miR-455-3p mimic and claudin-4-MUT (Fig. 2B). We subsequently transduced A549 cells with the NC, miR-455-3p mimics, or miR-455-3p inhibitors, and determined the effect of miR-455 on cellular proliferation and expression of claudin-4. The overexpression of miR-455-3p inhibited cell viability (*P* < 0.0001) and proliferation (*P* = 0.0072), and promoted apoptosis (*P* = 0.0017), while miR-455-3p inhibitors enhanced cell viability (P < 0.0001) and proliferation (*P* = 0.0027), and suppressed apoptosis (*P* = 0.0017) (Fig. 2C–E). The overexpression of miR-455-3p inhibited the mRNA and protein expression of claudin-4 while miR-455-3p inhibitors increased the expression levels of claudin-4 (Fig. 2F and G). The efficiency of miR-455 transfection was confirmed by qRT-PCR (Fig. 2H). It was additionally observed that the increase in the expression of miR-455 following treatment with LPS was reversed by allicin (Fig. 2I).

### 3.4 MiR-455-3p overexpression partially reversed the protective effect of allicin in LPS-treated A549 cells

**Fig. 3:**
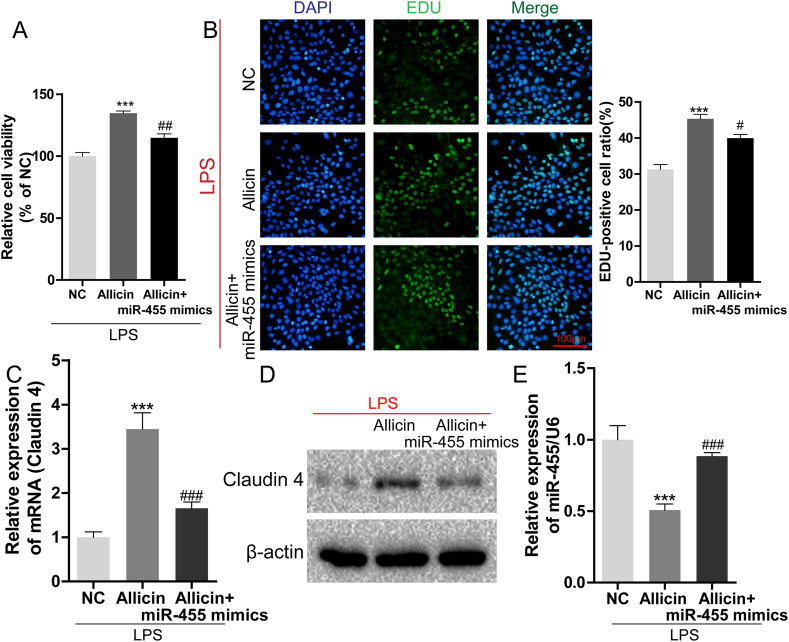
MiR-455 overexpression partially reversed the protective effect of allicin on LPS-treated A549 cells. (A) The viability of A549 cells was measured by CCK-8 assays. (B) The proliferation of A549 cells was detected by EdU assays (magnification: 200 × ). The expression of claudin-4 mRNA (C) and protein levels (D) in A549 cells were determined by RT-PCR and western blotting, respectively. (E) The expression of miR-455 in A549 cells was determined by qRT-PCR. ∗∗∗*P* < 0.001 vs. NS; #*P* < 0.05, ##*P* < 0.01, ###*P* < 0.001 vs. ALI. The original Western blot bands are shown in Fig. S4 in the supplemental file.

In order to further investigate whether allicin protects against LPS-induced injury by regulating miR-455-3p, miR-455-3p mimics were transfected into allicin-treated A549 cells. The results of CCK-8 assays revealed that cell viability was suppressed in the LPS + allicin + miR-455-3p mimics group compared to that of the LPS + allicin group (*P* = 0.003; Fig. 3A). The EdU assays demonstrated that the positive cell rate was decreased in the LPS + allicin + miR-455-3p group compared to that of the LPS + allicin group (*P* = 0.0295; Fig. 3B). The results of PCR and western blotting demonstrated that the expression levels of claudin-4 mRNA and protein were decreased in the LPS + allicin + miR-455-3p group compared to those of the LPS + allicin group (Fig. 3C and D). The expression patterns of miR-455-3p were the reverse of those of claudin-4 (Fig. 3E). These results indicated that the overexpression of miR-455-3p reversed the protective effect of allicin on ALI *in vitro*.

### 3.5 Allicin protected against LPS-induced ALI via the miR-455-3p/claudin-4 axis *in vivo*

**Fig. 4:**
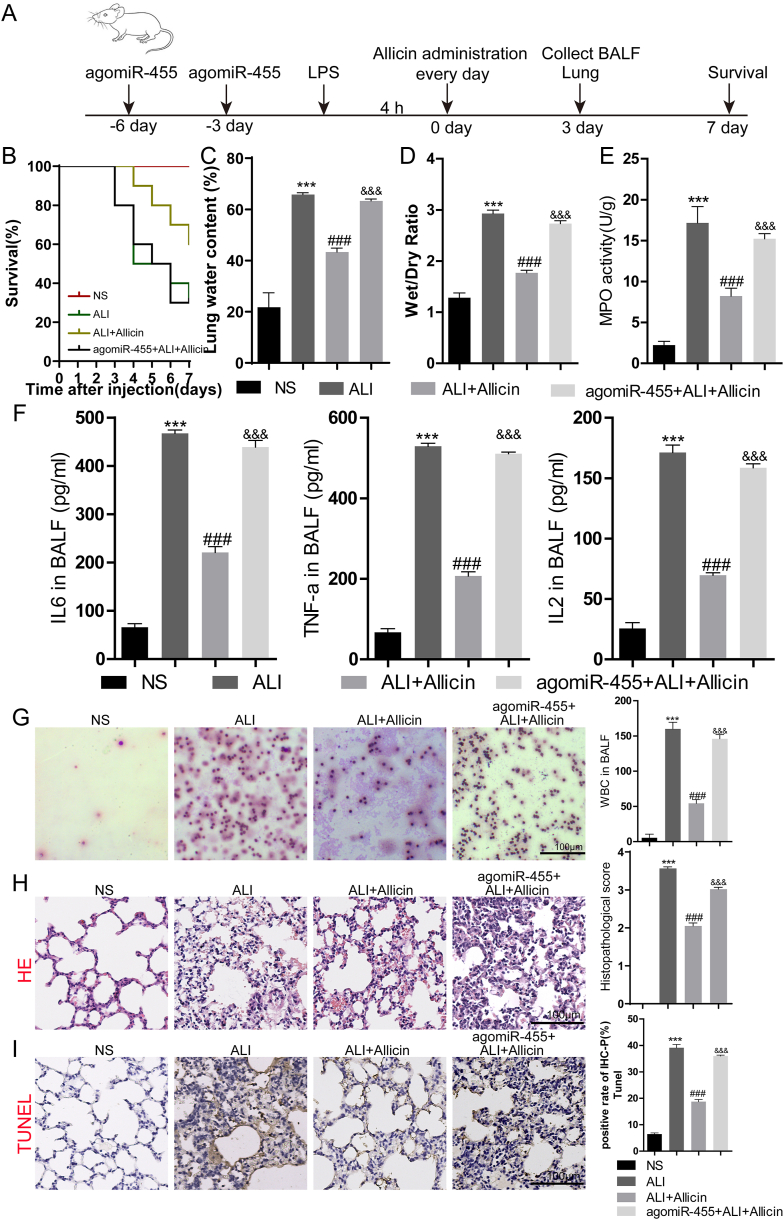
Allicin attenuated LPS-induced ALI *in vivo*. (A) Timeline of the study. (B) Kaplan-Meier survival curves. (C) Lung water content. (D) W/D ratio of lung weights. (E) MPO activity. (F) Levels of inflammatory factors in the BALF. (G) The number of white blood cells was counted by Wright's-Giemsa staining. (H) HE staining of lung sections. (I) TUNEL staining of lung sections. The values are presented as the mean ± SD (n = 3). ∗∗∗*P* < 0.001 vs. NS; ###*P* < 0.001 vs. ALI; &&& *P* < 0.001 vs. ALI + Allicin.

We subsequently investigated the effect of miR-455-3p *in vivo*. The timeline of the study is provided in Fig. 4A. As depicted in Fig. 4B, the survival rate of the ALI group was significantly reduced compared to that of the normal saline (NS) group. The survival rate of the group treated with allicin was improved compared to that of the groups with ALI, which was suppressed by agomiR-455 (Fig. 4B). Exposure to LPS increased the lung water content, the lung wet/dry (W/D) weight ratio, and activity of myeloperoxidase (MPO) compared to those of the rats in the NS group, and the effects of LPS were inhibited by the administration of allicin. However, agomiR-455 abolished the remissive effect of allicin on ALI (*P* < 0.0001; Fig. 4C–E). The levels of interleukin (IL)-6, tumor necrosis factor (TNF)-α, and IL-2, and the population of white blood cells were significantly reduced in the bronchoalveolar lavage fluid (BALF) of allicin-treated rats with ALI, which indicated that allicin attenuated pulmonary inflammation in rats with ALI. However, the therapeutic effects of allicin on ALI were reversed by agomiR-455 (*P* < 0.0001; Fig. 4F and G). Additionally, hematoxylin and eosin (HE) staining revealed that LPS induced histopathologic changes in the lung tissues, as indicated by the increased edema, thickness of the alveolar wall, and infiltration of inflammatory cells in the ALI group, which improved following treatment with allicin. However, agomiR-455 abolished the protective effect of allicin on ALI (*P* < 0.0001; Fig. 4H). The results of TUNEL assays revealed that the apoptosis of lung tissues in the group treated with allicin was significantly reduced compared to that of rats with ALI. However, the inhibitory effect of allicin on LPS-induced apoptosis was reversed by agomiR-455 (*P* < 0.0001; Fig. 4I).

**Fig. 5:**
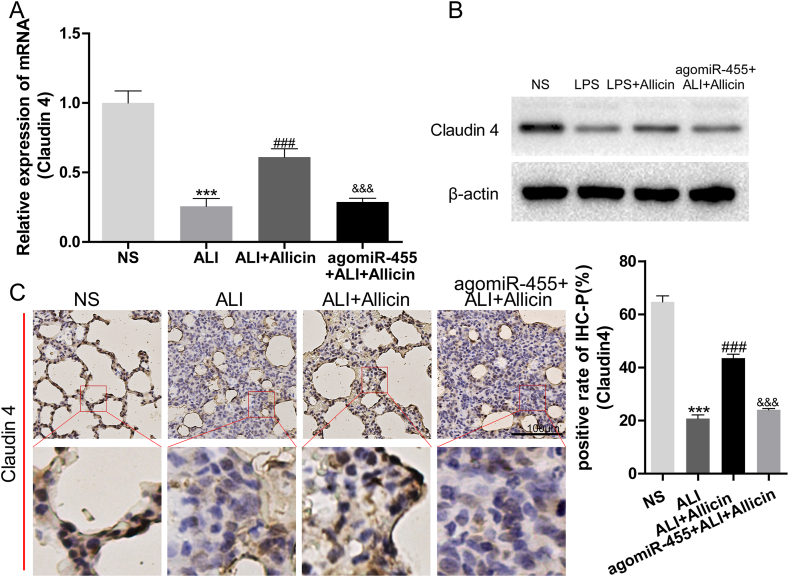
Treatment with allicin increased the expression of claudin-4 *in vivo*. (A) The expression of claudin-4 mRNA in the lung tissues was measured by RT-PCR. (B) Determination of the expression of claudin-4 protein in the lung tissues by western blotting. (C) Determination of the expression of claudin-4 in the lung tissues by IHC-P staining.∗∗∗*P* < 0.001 vs. NS; ###*P* < 0.001 vs. ALI; &&& *P* < 0.001 vs. ALI + Allicin. The original Western blot bands are shown in Fig. S5 in the supplemental file.

The expression of claudin-4 in the lung tissues of the aforementioned groups was determined by qRT-PCR, western blotting, and immunohistochemistry-paraffin (IHC-P) staining. The expression of claudin-4 mRNA and protein in the lung tissues of rats with ALI was markedly reduced following stimulation with LPS. Allicin restored the expression of claudin-4, which was reversed by the application of agomiR-455 (Fig. 5A and B). These findings were consistent with the results of IHC-P staining of claudin-4 (Fig. 5C). Altogether, the findings indicated that allicin protected against LPS-induced ALI by regulating the miR-455-3p/claudin-4 axis.

## 4 Discussion

The present study demonstrated that the protective effect of allicin in ALI was mediated via an increase in the levels of claudin-4 mRNA and protein in LPS-induced A549 cells. In order to elucidate the mechanism underlying the protective action of allicin, we predicted the interaction between miR-455-3p and claudin-4 using the TargetScan database, which revealed that claudin-4 was the downstream target of miR-455-3p. Additionally, the overexpression of miR-455-3p significantly attenuated the protective effect of allicin in LPS-induced A549 cells and rats with ALI. To the best of our best knowledge, this is study is the first to report that the protective effect of allicin on ALI is mediated via the regulation of the miR-455/Cldn4 axis. The findings provide further insights into the mechanism of action of allicin, and the study provides an experimental basis for the application of allicin in the treatment of ALI.

The protective effects of allicin in organ injury have been studied extensively in previous studies [32,33]. It has been reported that allicin reduces liver damage by inhibiting the activity of aminotransferase and alleviating liver injury [32]. Li et al. demonstrated that allicin has anti-apoptotic and antioxidative effects which restore renal function in renal I/R injury [33]. Allicin alleviates the damage to the intestinal epithelial barrier in rats with acrylamide-induced oxidative stress [34]. The findings of the present study were consistent with the results of our previous study and other studies [19,21], and confirmed the protective effects of allicin against LPS-induced ALI, which was indicated by the increased proliferation of LPS-induced A549 cells and the prolonged survival of rats exposed to LPS.

Numerous studies have demonstrated that miRNAs can inhibit cellular injury and protect against lung injury by targeting specific molecules, and are regarded as biomarkers and therapeutic targets of ALI [35,36]. In our previous study we demonstrated that allicin protects against LPS-induced ALI by upregulating the expression of claudin-4 [21]. Additionally, recent studies have reported that miRNAs play a role in the protective effect of allicin in I/R injury and tumors [16,37]. We therefore speculated that miRNAs could play a role in the regulation of claudin-4 expression by allicin. In this study, the prediction results of the TargetScan database revealed that only miR-455-3p binds to claudin-4. The association between miR-455-3p and claudin-4 was further confirmed by dual-luciferase reporter assays. Jiang et al. reported that hypoxia injury downregulates the expression of miR-455, and preconditioning with H_2_S attenuates the apoptosis of lung epithelial cells by upregulating the expression of miR-455 [38]. However, the present study revealed that LPS increased the expression of miRNA-455-3p and decreased the expression of claudin-4 in A549 cells, while treatment with allicin reversed the changes induced by LPS. In this study, we observed that pre-treatment with miR-455-3p mimics negated the protective effect of allicin. This indicated that allicin downregulated the expression of miRNA-455-3p, which induced claudin-4 overexpression and thereby protected against LPS-induced lung injury. This conjecture was subsequently confirmed by *in vivo* experiments.

The present study demonstrated the role of miR-455-3p in the reversal of LPS-induced ALI by allicin; however, this study has several limitations, which are described hereafter. Using the TargetScan database, we initially determined that only mir-455-3p binds to claudin-4; however, the role of other miRNAs in the regulation of claudin-4 cannot be excluded. Additionally, further studies are necessary for determining whether miR-455-3p regulates other target genes in ALI. Secondly, although the present study revealed that allicin can alleviate lung injury *in vivo*, further studies using larger animal models and sample sizes are necessary for validating this finding. It is also necessary to investigate the pharmacokinetics and pharmacodynamics of allicin in future studies.

## 5 Conclusions

This study is the first to demonstrate that miR-455-3p could be involved in the protective action of allicin against LPS-induced lung injury, and the most significant finding was that miR-455-3p binds to claudin-4. MiR-455-3p negatively regulates claudin-4, and miR-455-3p expression was downregulated in the group treated with allicin compared to that of the LPS-treated group. The finding suggesting that allicin has potential therapeutic value in the treatment of lung injury, and that its functions are mediated via the miR-455-3p/claudin-4 axis.

## CRediT authorship contribution statement

**Yueliang Zheng:** Resources, Funding acquisition, Data curation. **Gaoxiang Li:** Methodology, Investigation. **Aili Shi:** Software, Formal analysis. **Junping Guo:** Methodology. **Yingge Xu:** Data curation. **Wenwei Cai:** Writing – review & editing, Writing – original draft, Funding acquisition, Conceptualization.

## Ethics approval and consent to participate

All animal experiments were approved by the Ethics Committee of the First Affiliated Hospital of Zhejiang University.

## Consent to publication

All authors have given their consented to the publication of this manuscript.

## Data availability

Data are available upon reasonable request.

## Funding

This work was supported by the Project of Zhejiang Administration of Traditional Chinese Medicine [grant numbers 2020ZZ002, 2021ZZ003]; the 10.13039/501100004731Natural Science Foundation of Zhejiang Province [grant number LZ22H150001]; and the 10.13039/501100001809National Natural Science Foundation of China [grant number 82072161].

## Declaration of competing interest

The authors declare that they have no known competing financial interests or personal relationships that could have appeared to influence the work reported in this paper.
