# Supplementary material for: Role of miR-455-3p in the alleviation of LPS-induced acute lung injury by allicin
Source: Heliyon. 2024 Oct 12;10(20):e39338. doi: 10.1016/j.heliyon.2024.e39338 (PMC11535764; doi:10.1016/j.heliyon.2024.e39338)
Supplement: Multimedia component 1 [file mmc1.docx]

**Role of miR-455-3p in the alleviation of LPS-induced acute lung injury by allicin**

**Running title:** MiR-455-3p mediates the protective effect of allicin in ALI

Yueliang Zheng^1^, Gaoxiang Li^1^, Aili Shi^1^, Junping Guo^2^, Yingge Xu^1^, Wenwei Cai^1,*^

^1^ Emergency and Critical Care Center, Department of Emergency Medicine, Zhejiang Provincial People's Hospital (Affiliated People's Hospital), Hangzhou Medical College, Hangzhou, Zhejiang, China

^2^ Rainbowfish Rehabilitation & Nursing School, Hangzhou Vocational & Technical College, Hangzhou, Zhejiang, China.

*Correspondence to:

Wenwei Cai, Emergency and Critical Care Center, Department of Emergency Medicine, Zhejiang Provincial People's Hospital (Affiliated People's Hospital), Hangzhou Medical College, Hangzhou, Zhejiang, China, 158 Shangtang Road, Hangzhou City, Zhejiang Province, 310014, China; Email: wwcai@139.com.


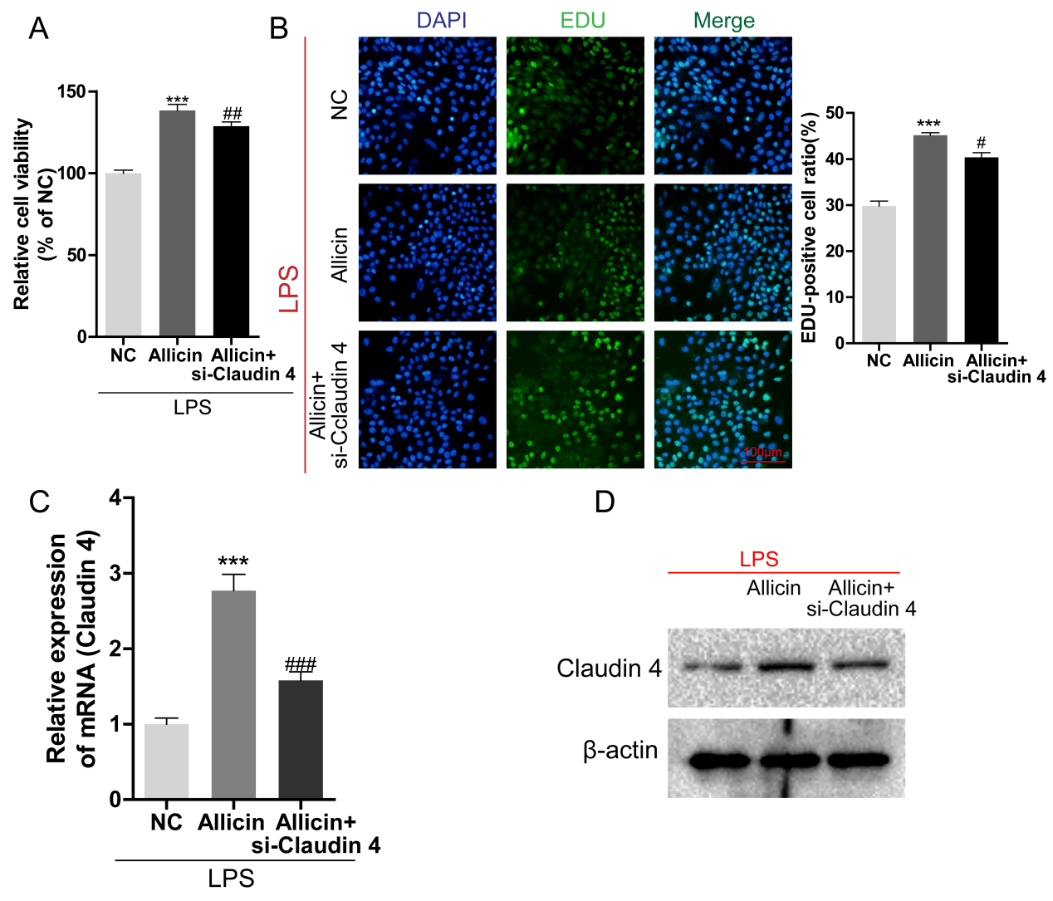


**Figure S1** Allicin protects against LPS-induced injury in A549 cells by up-regulating Claudin-4. (A) CCK-8 assay was used to measure the viability in each group as indicator. (B) EdU assay was used to measure the cell proliferation in each group as indicator. The Claudin-4 mRNA expression (C) and protein level (D) was determined by RT-PCR and Western blot, respectively. The original Western blot bands are shown in Fig. S6 in the supplemental file.

**Actin Claudin-4**







**Figure S2 original blot**

**Actin Claudin-4**







**Figure S3 original blot**

**Actin Claudin-4**







**Figure S4 original blot**

**Actin Claudin-4**




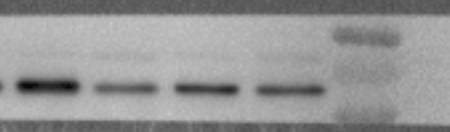


**Figure S5 original blot**

**Actin Claudin-4**


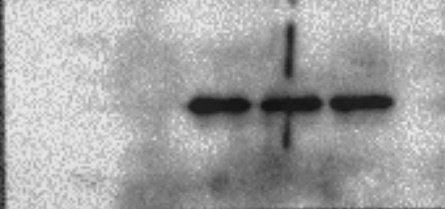

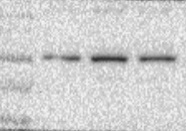


**Figure S6 original blot**
